# Shared decision making for stroke prevention in atrial fibrillation: study protocol for a randomized controlled trial

**DOI:** 10.1186/s13063-017-2178-y

**Published:** 2017-09-29

**Authors:** Marleen Kunneman, Megan E. Branda, Peter A. Noseworthy, Mark Linzer, Bruce Burnett, Sara Dick, Gabriela Spencer-Bonilla, Cara A. Fernandez, Haeshik Gorr, Mike Wambua, Shelly Keune, Claudia Zeballos-Palacios, Ian Hargraves, Nilay D. Shah, Victor M. Montori

**Affiliations:** 1 Knowledge and Evaluation Research Unit, Division of Endocrinology, Diabetes, Metabolism and Nutrition, Department of Medicine, Mayo Clinic, 200 First Street SW, Rochester, MN 55905 USA; 2 Health Services Research, Mayo Clinic, Rochester, MN USA; 3 Robert D. and Patricia E. Kern Center for the Science of Health Care Delivery, Mayo Clinic, Rochester, MN USA; 4 Heart Rhythm Section, Department of Cardiovascular Diseases, Mayo Clinic College of Medicine, 200 First Street SW, Rochester, MN 55905 USA; 5 Division of General Internal Medicine, Hennepin County Medical Center, Minneapolis, MN USA; 6 Thrombosis Clinic and Anticoagulation Services, Park Nicollet Health Services, St Louis Park, MN USA; 7 University of Minnesota and Minneapolis Medical Research Foundation, Minneapolis, MN USA; 8 School of Medicine, University of Puerto Rico Medical Sciences Campus, San Juan, USA; 9 Division of Health Care and Policy Research, Mayo Clinic, Rochester, MN USA

**Keywords:** Atrial fibrillation, Anticoagulation, Shared decision making, Decision aid, Conversation aid, communication, Medication uptake, Medication adherence

## Abstract

**Background:**

Nonvalvular atrial fibrillation (AF) is a common ongoing health problem that places patients at risk of stroke. Whether and how a patient addresses this risk depends on each patient’s goals, context, and values. Consequently, leading cardiovascular societies recommend using shared decision making (SDM) to individualize antithrombotic treatment in patients with AF. The aim of this study is to assess the extent to which the Anticoagulation Choice conversation tool promotes high-quality SDM and influences anticoagulation uptake and adherence in patients with AF at risk of strokes.

**Methods:**

This study protocol describes a multicenter, encounter-level, randomized trial to assess the effect of using the Anticoagulation Choice conversation tool in the clinical encounter, compared to usual care. The participating centers include an academic hospital system, a suburban community group practice, and an urban safety net hospital, all in Minnesota, USA. Patients with ongoing nonvalvular AF at risk of strokes (CHA_2_DS_2_-VASc score ≥ 1 in men, or ≥ 2 in women) will be eligible for participation. We aim to include 999 patients and their clinicians. The primary outcome is the quality of SDM as perceived by participants, and as assessed by a post-encounter survey that ascertains (a) knowledge transfer, (b) concordance of the decision made, (c) quality of communication, and (d) satisfaction with the decision-making process. Recordings of encounters will be reviewed to assess the extent of patient involvement and how participants use the tool (fidelity). Anticoagulant use, choice of agent, and adherence will be drawn from patients’ medical and pharmacy records. Strokes and bleeding events will be drawn from patient records.

**Discussion:**

This study will provide a valid and precise measure of the effect of the Anticoagulation Choice conversation tool on SDM quality and processes, and on the treatment choices and adherence to therapy among AF patients at risk of stroke.

**Trial registration:**

ClinicalTrials.gov, NCT02905032. Registered on 9 September 2016.

**Electronic supplementary material:**

The online version of this article (doi:10.1186/s13063-017-2178-y) contains supplementary material, which is available to authorized users.

## Background

Atrial fibrillation (AF) is the most common sustained cardiac arrhythmia, affecting approximately 3 million people in the USA [1, 2], and accounting for $26 billion in healthcare costs annually [3]. AF-related thromboembolic strokes are often devastating and can impose a substantial physical, social, and economic burden [4–7]. The risk of stroke can be reduced by about 68% with anticoagulation with vitamin K antagonists (VKAs) such as warfarin [8–13]. Pivotal trials have shown that direct oral anticoagulants (DOACs) have equivalent or superior efficacy and safety to warfarin [14–16]. Furthermore, these agents are associated with a lower risk of intracranial bleeding than warfarin and the considerable advantages of reduced dietary restrictions, drug-drug interactions, and need for ongoing blood draws for monitoring. With the introduction of this new class of medications, patients and providers now have substantially greater choices for stroke prevention in AF.

Nonetheless, underuse of anticoagulation continues to be a significant quality gap. Despite patients’ strong desire to prevent strokes [17, 18], less than half of high-risk patients with AF receive anticoagulants [19], and of those who start anticoagulation, 30–50% stop treatment within 12 months [20–23]. This underuse of anticoagulation likely stems, at least in part, from patient and clinician concerns about anticoagulation-related bleeding [19–24]. It also suggests that some patients cannot implement anticoagulation in their lives: warfarin requires a stable diet and periodic laboratory (international normalized ratio (INR)) monitoring [25–27], while DOACs are costly and bleeding reversal agents are not readily available [14–16]. Underuse may also result from poor patient and clinician access to, and deliberation with, individualized estimates of risks and benefits [28, 29]. Consequently, patients and clinicians require support in initiating and implementing appropriate anticoagulation therapy.

In 2014, three major cardiovascular organizations formulated guidelines for the management of patients with AF, giving their strongest class I recommendation for using shared decision making (SDM) to individualize anticoagulation in patients with AF at risk of strokes [30]. In SDM, patients and clinicians work together to make decisions about reasonable anticoagulation strategies matched to medical risk and patient circumstance [30–32]. However, translating this recommendation for SDM into practice is challenging. The guideline provides no guidance on how to achieve SDM, and there are no up-to-date validated tools to support SDM in this context. Furthermore, the effect of SDM on anticoagulation rates and adherence in patients with AF is unknown [30].

To implement the 2014 guidelines recommendation in usual practice, we developed Anticoagulation Choice. This tool was designed to promote an SDM conversation in the clinical encounter between two experts: the clinician, who is expert in medical issues, and the patient, who is expert on how anticoagulation may fit their life and context, issues that bear on adherence. In previous research in other clinical contexts, we have demonstrated the practical impact of SDM conversation tools [33–36]. We aimed to assess the extent to which using the Anticoagulation Choice tool promotes high-quality SDM and impacts anticoagulation uptake (initiation) and adherence in patients with AF at risk of strokes. In this manuscript, we describe the methods and protocol we will use to test the effectiveness of Anticoagulation Choice.

## Methods

### Study design

This multicenter, encounter-level, randomized trial compares the impact of usual care or clinicians’ use of the Anticoagulation Choice tool on SDM and anticoagulation use outcomes in patients with AF at risk of thromboembolic strokes. This tool is designed to support SDM about whether and how to perform anticoagulation to reduce stroke risk. Institutional Review Board approval has been obtained from the lead coordinating center, the Mayo Clinic (approval number 16-005409) and from the two participating external sites. The trial is registered at ClinicalTrials.gov (registration number NCT02905032, date of registration 9 September 2016). Our protocol adheres to the standard protocol items: recommendation for interventional trials (SPIRIT) recommendations (see Additional file 1) [37].

### Study setting

The clinical trial will take place at three hospital sites in Minnesota that treat patients with AF: an academic medical center, a suburban community group practice, and an urban safety-net health system.

### Eligibility criteria

All clinicians – physicians, nurse practitioners, physician assistants, and pharmacy doctors – that have conversations about anticoagulation with patients with AF at the participating sites are eligible for participation.

Patients are eligible if they are adults (18 years of age or older) with nonvalvular AF (hemodynamically significant mitral stenosis or mechanical valve replacement) deemed at high risk of thromboembolic strokes (CHA_2_DS_2_-VASc score ≥ 1 in men, or ≥ 2 in women), who, during the consent process, are deemed (a) able to read and understand the informed consent document as determined by the study coordinator and (b) a candidate for anticoagulation by the patient’s clinician.

We will create two cohorts of patients for descriptive and analytical purposes. The first cohort (“start” cohort) consists of patients who are not ongoing users of an anticoagulant at study enrollment. They may have used anticoagulation and discontinued > 6 months ago, never used anticoagulation, or take aspirin only. The second cohort (“review” cohort) consists of patients who, within 6 months of study enrollment, took or are taking warfarin or DOACs, but may reconsider their current approach. Examples of patients who may be a part of this cohort may include patients who have difficulty maintaining a therapeutic INR, or patients considering switching to a different anticoagulant.

### Participant identification and recruitment

Eligible clinicians will be recruited through presentations at department meetings and just in time prior to a scheduled appointment with an eligible patient. Clinicians must provide written informed consent for study participation prior to enrolling their first patient.

**Fig. 1:**
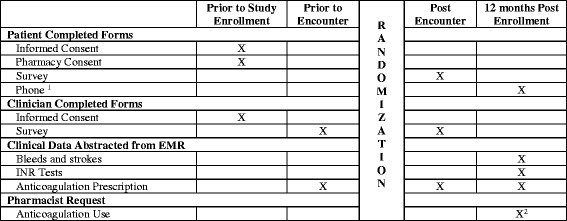
Schedule of enrollment, interventions and assessments. Notes: (1) patients who do not have utilization within enrolling healthcare system will be contacted via phone for verification of safety data (strokes and bleeds). If there is no information in the record and follow up is necessary we will call patients the maximum number allowed by the IRB followed by a postal survey; (2) pharmacist records will be requested for 12 months prior to enrollment through 10 months post enrollment. INR international normalized ratio, EMR electronic medical record

Eligible patients will be identified through appointment lists for patients with AF in primary care, cardiology, neurology, thrombophilia and anticoagulation clinics, electrocardiogram (ECG) result lists, medical records, and clinical referrals. Eligible patients will be recruited through phone calls (asking patients to arrive early to their scheduled appointment to complete the consent process), or in person at the time of their scheduled appointment. Patients will be consented in person, in a private location (e.g., clinic/exam room, or hospital room) prior to their appointment. The study coordinator will share study information with patients, answer their questions, and obtain their written informed consent. In the spirit of minimally disruptive research [38], all study activities will occur within scheduled appointments, avoiding the need for additional research-only visits (Fig. 1). Study information and consent forms for patients and clinicians can be found in Additional files 2 and 3.

### Randomization and blinding

Eligible patients will be allocated into either use the Anticoagulation Choice tool (intervention) arm or the care-as-usual arm. A random sequence, generated a priori by the trial statistician through a computer generated system, will be used for 1:1 allocation stratified by clinic (academic, community or safety net), cohort (start or review), and stroke risk (CHA_2_DS_2_-VASc score of ≥ 1 for men and ≥ 2 for women) using blocks of random size. Study coordinators will allocate patients at time of consent by accessing the REmote Data Capture (REDCap) system [39]. Except for patients, who will be informed that the trial will be testing different ways clinicians and patients with AF communicate about anticoagulation, all study personnel will be able to discern participant allocation.

### Intervention

In the intervention group, clinicians will conduct the encounter as usual with access to the Anticoagulation Choice tool (see Additional file 4, [40]). The tool consists of two components: a risk calculator to calculate personalized risks for thromboembolic strokes at 1 and 5 years, and issue cards that support the patient-clinician conversation on patient-important factors that may affect the choice of agent and patient ability to adhere to anticoagulation (e.g., diet, recreational activities, and travel).

The tool will be accessed online or through an available link in the electronic medical record (EMR). Patient information to complete the calculations of risk of stroke (CHA_2_DS_2_-VASc [41]) and bleeding (HAS-BLED [42]) will be verified by the clinician and entered into the tool. Patients can request a printed copy of the tool from their clinician which they can use later to share with others, and to review, confirm or revisit the decision.

In the care-as-usual group, clinicians will conduct the encounter per their standard care. To ensure contamination does not occur, the study coordinator will inform the clinician prior to entering the room that the patient is to receive standard care and that the tool is not to be accessed.

#### Training

Participating clinicians at each study site will complete brief training on the use of the tool either alone or in a group setting. Study staff will train all clinicians with an interactive demonstration of the tool guided by a semi-structured protocol, and, when opportune, brief video clips demonstrating the use of the conversation tool in a simulated encounter. During training, clinicians will be reminded that the tool is not a script, and that it should be used to support not replace the conversation in a manner that makes sense in their practice. Study staff will be available to answer questions about the use of the tool or to re-train clinicians when deviations in the quality of delivery are observed.

### Data collection and management

Patients that agree to participate will be captured in the REDCap system [39]. We will document in a recruitment tracking log all potentially eligible patients found to be ineligible or who decline participation. The reason for ineligibility or reason for decline will be captured along with age, sex, race, and ethnicity.

At the time of their enrollment, clinicians will complete a demographic survey. After each encounter, study staff will administer clinicians and patients a survey. If patients request a return envelope, one will be provided to return the survey by mail. If the survey is not received in the 10 days post encounter, a reminder will be mailed to them with a copy of the survey along with a return envelope. A courtesy call will be made within 5 days post mailing. Every effort will be made to complete the survey at the clinic immediately after completion of the clinical encounter as this offers the best chance for complete data collection. Post-encounter questionnaires for patients and clinicians can be found in Additional files 5 and 6.

Data from the medical record will be abstracted for all enrolled patients to capture demographic, clinical, and medication prescription data. The time frame for collection will be from prior to enrollment to 12 months post enrollment. For patients that do not have any encounters at the institution in the 12 months post enrollment, a scan will be conducted up to 6 months after the 12-month timeline to verify continuity of care at the institution, change in contact information and/or survival status. If no records are available at that time, we will call the patient (number of attempts as authorized by each IRB), followed by a postal survey if nonresponse persists.

### Measures and outcomes

#### Participant characteristics

Through a self-report post-encounter questionnaire, we will collect patients’ socio-demographic information, residency (e.g., home or nursing home), location of primary healthcare, medications, history of bleeding, alcohol use, a major fall, or medical conditions that increase risk of bleeds. These data include variables necessary to estimate the risk of stroke and bleeding. To further characterize the patients, we will use a single-item health literacy screener from Chew et al. [43, 44], a four-item modified version of the Subjective Numeracy Scale [45], and a single-item health status measure [46]. Through medical record review, we will collect information on past use of anticoagulants.

The post-encounter survey for clinicians will collect demographic and work-related data, such as age, gender, specialty, percentage of their practice dedicated to anticoagulation care, and two single-item measures of emotional exhaustion and depersonalization (burnout) [47].

#### Participant reported outcomes

The primary outcome being assessed is the quality of SDM as perceived by the participants, which is a multidimensional construct. This endpoint is assessed through (a) knowledge transfer, (b) concordance, (c) quality of communication, and (d) satisfaction with the decision-making process.

Knowledge transfer is assessed through six questions about AF and anticoagulation. In an additional question, patients’ knowledge of their own risk of stroke will be assessed by asking them to provide a value between 0 and 100.

Concordance between the clinician and the patient about what was decided will be determined by comparing their reported course of action, including stop taking, starting, continuing, or not starting an anticoagulant.

The quality of communication will be assessed with a modified version of three questions from the CAHPS Clinician and Group survey [48]. These questions indicate the extent to which the communication is patient-centered, covering technical (explain things in a way you could understand) and affective (show respect for what you have to say) communication.

Patient decision satisfaction will be assessed using the Decisional Conflict Scale, reflecting the degree of uncertainty about the choice [49, 50]. High scores on this scale are associated with delay in acting on the decision. Both patient and clinician satisfaction will be assessed with a single-item question on a 7-point Likert scale that addresses whether they would recommend the approach used to others (other patients or clinicians, respectively). The clinician will also be asked to indicate their satisfaction with the discussion about anticoagulation on a 5-point Likert scale.

#### Encounter outcomes

With consent from participants, we will record clinical encounters. The extent of SDM during the encounter will be assessed by using the OPTION12-scale, an observer reviewer scale coding the degree of patient involvement by the clinician [51]. The reproducibility, validity, and responsiveness of the OPTION12-scale have been shown to be adequate [52]. Despite these features, we will not use assessments from the recordings as primary endpoints, as the proportion of encounters without video or audio could threaten the validity, and it is impossible to blind the reviewers to allocation, increasing the risk of bias. The length in minutes of the discussion about anticoagulation will be assessed. In addition, observers will review recordings to describe how the tool is used during the encounter (referred to as “fidelity”, e.g., which parts of the tool are used, (how) were risks discussed, which options were discussed, were the issues of greatest salience to the patient chosen to discuss). Using the same approach in the control arm, will enable us to assess potential contamination between arms.

#### Medical outcomes

Ten months post enrollment, we will review medical and pharmacy records to assess the rate of anticoagulation use, choice of anticoagulant, and clinical events. Deaths, strokes and transient ischemic events requiring medical assistance, and bleeding episodes requiring medical assistance will be noted. We will calculate anticoagulation persistence using the proportion of days covered (PDC) at 12 months [53]. We will also review all pharmacy refills for the 12 months prior to enrollment, which will allow us to calculate persistence for review cohort patients to compare to persistence post encounter [53]. For patients who choose to stay on or start warfarin, we will use as secondary measures of adherence the proportion of INR tests obtained/scheduled and the percentage of time at therapeutic target (typically INR 2–3) through medical record review.

### Statistical considerations

#### Sample size

Table 1 shows the detectable effect for each of the outcomes of interest if we were to have data on that outcome from a total of 333 patients, a third of the total population we plan to recruit. This provides enough power (α = 0.05; two-sided difference) to detect meaningful differences across arms for all SDM quality and process outcomes. Our intent, however, is to have enough power to detect important differences when we conduct analyses of groups or cohorts of patients. Most of these analyses will be performed with the participants divided into two cohorts (e.g., start and review cohorts), except for the analyses by clinic, in which the total sample will be divided into three cohorts (of about 333 per cohort): academic, community, and safety-net clinic. To address all planned subgroup analyses, we would need three times the sample size listed in the table, or 999 participants, assuming even distribution of participants per subgroup.

**Table 1:** Detectable effect for each of the outcomes of interest

Outcome (n = 333)	Rate (%) or SD	Detectable effect	Power*
Patient level – SDM quality			
Knowledge transfer^a^	18	5.6	84%
Knowledge of risk	55%	15%	81%
Decisional conflict scale^a^	17	5.2	80%
Clinician level			
Satisfaction^a^	54%	15%	80%
Encounter level – SDM process			
Engagement (OPTION12)^a^	12.6	3.9	80%

^a^Values from iADAPT shared decision making (SDM) tool trial (reference)

*α = 0.05; two-sided

We expect approximately 90% of patients to receive a prescription to start or continue a medication. Of those, we can reasonably expect to obtain > 85% of the pharmaceutical records, which will be requested of all enrolled patients regardless of decision. Using the estimated trial size of 999 participants, we will have approximately 765 patient records available for assessment of anticoagulant persistence using the PDC at 12 months. In our review of the Optum database, 40% of patients were adherent to anticoagulation (defined as > 80% PDC, the threshold used by Centers for Medicare & Medicaid) at 12 months. Assuming an expected rate of 60% PDC for the usual care cohort, we would have 80% power to detect a 9% difference (69% PDC in the Anticoagulation Choice tool arm), with a two-sided test and an alpha of 0.05. In subgroup analyses comprising 100 participants per arm and using a one-sided test and alpha of 0.05, we will have power to detect differences of at least 16%.

#### Analysis plan

The study will be analyzed according to the intention-to-treat principle, including all patients enrolled to the study in the arm to which they were randomly assigned. Full reports will include cohorts with complete data and intention-to-treat cohorts in which imputation methods will be used to address missing data. Baseline characteristics will be reported in the study results with continuous values being reported as means and standard deviations and categorical values reported as counts and frequencies. Any baseline imbalances (*p* < 0.05) will be explored as possible factors to adjust main analyses. We will adhere to the CONSORT guidelines to report all trial results.

We will test differences between arms using *t* tests for continuous outcomes and chi-square tests for dichotomous outcomes. If there are differences in baseline characteristics found by statistical means or found to have clinical relevance between the two study arms, these will be accounted for using regression models, which will include an indicator for study arm.

We will describe any potential heterogeneity of treatment effect reporting subgroup results to facilitate synthesis of subgroup results in future meta-analyses. We will assess heterogeneity of treatment effect by clinic (academic, community and safety net), by cohort (start or review cohort), by stroke risk (CHA_2_DS_2_-VASc score ≥ 1 in men, or 2 in women), and by numeracy (less than adequate versus not) for all SDM trial outcomes.

We will not assume that SDM outcomes are independent of clinician, but rather test to see if patients seen by the same clinician have correlated outcomes. Ignoring such clustering effects would result in over-narrow confidence intervals and potentially false positive study results. Instead, if clustering is seen, determined by calculating the intra-class correlation (ICC > 0.05) for each outcome, then the value for the ICC will be reported in findings. We will use cluster (at clinician level) adjusted *t* test and chi-square test for comparisons between arms and hierarchical generalized linear models with random main effects specified at the clinician level when adjusting by more than arm [54]. If clustering is not present then the results will reduce to a model that assumes independence and reflect findings appropriately.

#### Missing data

We will make every effort to minimize missing data. Trial enrollment and the fidelity of follow-up procedures will be reviewed during bi-weekly conference calls. A study biostatistician will conduct frequency reports to assess for missing data, and the study team will troubleshoot any problems encountered. We will report rates of missing data for each outcome by study arm and send missing data reports to sites.

#### Safety and monitoring

The trial itself is not expected to pose any medical risk to participants. Strokes and bleeding events requiring medical assistance will be monitored and reported to the data safety monitoring board. We will rely on a passive approach based on patient and clinician self-report, and on medical record review 12 months post enrollment. Should a patient not have healthcare utilization in the 3 months prior to the 12-month date, then the patient will be contacted directly to confirm that no stroke or bleeding episode requiring medical attention has taken place. If one has taken place, we will request authorization to obtain medical records from the facilities that took care of the patient for these events.

A data safety and monitoring plan and charter have been formed to monitor participant safety, data completeness and adherence to the study protocol; a board will meet bi-annually to review study reports prepared by the study statistician. The trial poses no potential harm for the patient as the intervention being tested in comparison to standard care is to create a conversation to assist in decision making. Thus, the trial will not incorporate an interim analysis, nor will an early stopping rule be put into place. The principal investigator, each of the site investigators, study statistician, and project coordinator will meet monthly to assess recruitment (overall and by site), baseline comparability of treatment groups, protocol adherence, completeness of data collection, safety, and fidelity of follow-up procedures. Unexpected adverse patient events related to the study or its procedures will be logged and reported to all IRBs overseeing the trial.

#### Patient Advisory Group

The Patient Advisory Group of the Mayo Clinic Shared Decision Making National Resource Center maintains a partnership with the Knowledge and Evaluation Research Unit in providing feedback on research proposals, participant recruitment materials, surveys, and all areas of proposed and existing research [55]. They view our research through the patient’s perspective, assisting us in detecting potential barriers, contributing to effective and meaningful research, and minimizing the footprint of research on patients’ lives. As with our other projects, we will collaborate with the Patient Advisory Group to improve our project and remain connected with the real world of the patients that will be impacted.

## Discussion

We have described the methods we will employ to assess the extent to which the Anticoagulation Choice conversation tool can promote high-quality SDM and affect anticoagulation uptake and adherence in patients with AF at risk of stroke. To this end, we will conduct a multicenter, patient-level, randomized trial comparing care as usual with and without usage of the tool.

Our study responds to the call from cardiovascular organizations for using SDM to individualize anticoagulation in patients with AF at risk of stroke [30]. Currently, no tools are available that are both up-to-date and proven to support SDM in this context, nor is the effect of SDM on anticoagulation rates and adherence in these patients known. Our study takes a first step in implementing the 2014 guidelines recommendation [30], by testing the effect of the Anticoagulation Choice, which was designed to promote an SDM conversation in the clinical encounter between the clinician and the patient. Based on earlier experiences in other clinical contexts [33–36], we anticipate that the use of the tool will equip clinicians and patients to exchange information about possible options available, including risk information, and information exchange about what matters most to patients.

Limited clinician engagement is the biggest threat to patient recruitment. As in our prior trials, we will enroll and retain clinicians using techniques of academic detailing by the research team. We aim for maximal data collection while imposing the smallest footprint on clinical care activities (minimally disruptive research) [38]. We will not use financial incentives, as clinicians often manifest intrinsic motivation to test SDM (i.e., “reminds me of why I went into medicine”). This justifies our decisions to randomize at the encounter level, ensuring that all participating clinicians will have a chance to experience SDM. There is a possible risk of contamination; however, when patients are randomized in the usual-care arm, clinicians will not be provided access to the tool, and possible contamination will be assessed by determining what clinicians do in usual-care encounters using the same checklist we are using to determine fidelity in the intervention encounters.

We anticipate that new evidence about existing anticoagulants and new options will emerge in the course of this study. The flexible conversation tool we are testing can accommodate new information without requiring changes in the trial design. We will document these changes and conduct sensitivity analyses to study what, if any, effects these changes may have caused.

Ultimately, our study will aim to produce a valid and precise measure of the effect of the Anticoagulation Choice conversation tool on SDM quality and processes, including estimates of intervention subgroup interactions across key subgroups. In addition, we will describe, for the first time, the distribution of the rates and choices of anticoagulation in a population of patients receiving usual care and SDM and estimate the impact that the use of our conversation tool will have on patient adherence to their decision. The findings of this trial will be made promptly, completely, and broadly available, and as with our other SDM conversation tools, the Anticoagulation Choice will be made freely available in the website of the Mayo Clinic Shared Decision Making National Resource Center, supporting our commitment to open and universal access [56]. To date, our conversation tools are used every four minutes, having touched over 200,000 lives worldwide.

## Trial status

Clinician enrollment started January 2017, patient enrollment started February 2017. We anticipate enrollment will be completed by March 2019.

## Additional files

Additional file 1: SPIRIT checklist. (PDF 129 kb)

Additional file 2: Patient study information and informed consent form. (PDF 129 kb)

Additional file 3: Clinician study information and informed consent form. (PDF 160 kb)

Additional file 4: The Anticoagulation Choice conversation tool. This conversation tool is an online tool intended for use by a patient and clinician together in a clinical encounter in which they are discussing how to respond to the patient’s risk of stroke due to nonvalvular atrial fibrillation (AF). The tool is intended to provide a supportive structure and information that patient and clinician may draw on during clinical conversations in various settings e.g. primary care, emergency department, cardiology and other specialty practices. Details on the full developmental process of the ANTICOAGULATION CHOICE conversation tool is described elsewhere (Hargraves et al, The Lion and the Snake: Design in Support of Shared Decision Making in Atrial Fibrillation. Manuscript in preparation). (PDF 742 kb)

Additional file 5: Patient post-encounter survey. (PDF 1449 kb)

Additional file 6: Clinician post-encounter survey. (PDF 42 kb)

## Abbreviations

AF: Atrial fibrillation
BMI: Body mass index
CAHPS: Consumer Assessment of Healthcare Providers and Systems
DOAC: Direct oral anticoagulant
HIPAA: Health Insurance Portability and Accountability Act
INR: International normalized ratio
IRB: Institutional review board
PDC: Proportion of days covered
SDM: Shared decision making
VKAs: Vitamin K antagonists
