# Supplementary material for: Shared decision making for stroke prevention in atrial fibrillation: study protocol for a randomized controlled trial
Source: Trials. 2017 Sep 29;18:443. doi: 10.1186/s13063-017-2178-y (PMC5622521; doi:10.1186/s13063-017-2178-y)
Supplement: Supplementary file 2 — Patient study information and informed consent form. (PDF 129 kb) [file 13063_2017_2178_MOESM2_ESM.pdf]

MAYO CLINIC

Name and Clinic Number

Approval Date: **January 18, 2017**
Not to be used after: **August 30, 2017**

RESEARCH PARTICIPANT CONSENT AND PRIVACY AUTHORIZATION FORM

Study Title: Shared Decision Making for Stroke Prevention in Atrial Fibrillation (SDM4Afib) (Patient)

IRB#: 16-005409

Principal Investigator: Peter A. Noseworthy, M.D. and Colleagues

Please read this information carefully. It tells you important things about this research study. A member of our research team will talk to you about taking part in this research study. If you have questions at any time, please ask us.

Take your time to decide. Feel free to discuss the study with your family, friends, and healthcare provider before you make your decision.

To help you decide if you want to take part in this study, you should know:

- Taking part in this study is completely voluntary.
- You can choose not to participate.
- You are free to change your mind at any time if you choose to participate.
- Your decision won't cause any penalties or loss of benefits to which you're otherwise entitled.
- Your decision won't change the access to medical care you get at Mayo Clinic now or in the future if you choose not to participate or discontinue your participation.

For purposes of this form, Mayo Clinic refers to Mayo Clinic in Arizona, Florida and Rochester, Minnesota; Mayo Clinic Health System; and all owned and affiliated clinics, hospitals, and entities.

If you decide to take part in this research study, you will sign this consent form to show that you want to take part. We will give you a copy of this form to keep.

If you are signing this consent form for someone else, "you" in the consent form refers to the participant.

MAYO CLINIC

Name and Clinic Number

Approval Date: **January 18, 2017**
Not to be used after: **August 30, 2017**

CONTACT INFORMATION

You can contact …	At …	If you have questions about …
**Principal Investigator:**<br>Dr. Peter A. Noseworthy<br><br>**Study Team Contact:**<br>Cara Fernandez	**Phone:**<br>(507) 293-0175<br><br>**Phone:**<br>(507) 266-1897<br><br>**Institution Name and Address:**<br>Mayo Clinic<br>200 First Street SW<br>Rochester, MN 55905	▪ Study tests and procedures<br>▪ Research-related injuries or emergencies<br>▪ Any research-related concerns or complaints<br>▪ Withdrawing from the research study<br>▪ Materials you receive<br>▪ Research-related appointments
**Mayo Clinic Institutional Review Board (IRB)**	**Phone:**<br>(507) 266-4000<br><br>**Toll-Free:**<br>(866) 273-4681	▪ Rights of a research participant
**Research Subject Advocate (The RSA is independent of the Study Team)**	**Phone:**<br>(507) 266-9372<br><br>**Toll-Free:**<br>(866) 273-4681<br><br>**E-mail:**<br>researchsubjectadvocate@mayo.edu	▪ Rights of a research participant<br>▪ Any research-related concerns or complaints<br>▪ Use of your Protected Health Information<br>▪ Stopping your authorization to use your Protected Health Information
**Research Billing**	**Rochester, MN:** (507) 266-5670	▪ Billing or insurance related to this research study

A description of this clinical trial will be available on http://www.ClinicalTrials.gov, as required by U.S. Law. This Website will not include information that can identify you. At most, the Website will include a summary of the results. You can search this Website at any time.

1. Why are you being asked to take part in this research study?

We are inviting you to be a part of this research study because you have an appointment with your clinician to discuss treatments for atrial fibrillation.

2. Why is this research study being done?

This research study is being done to develop educational materials that will help patients and clinicians discuss atrial fibrillation and options to treat it.

3. Information you should know

Who is Funding the Study?

The National Institute of Health (NIH) is funding the study. The NIH will pay the institution to cover costs related to running the study.

Information Regarding Conflict of Interest:

No investigators have reported a conflict of interest with this study.

4. How long will you be in this research study?

Your active participation will last until you complete the survey following your appointment today. Also, there is a chance you could be contacted approximately 10 months after enrollment to verify pharmacy records.

5. What will happen to you while you are in this research study?

If you agree to participate, you will be asked to participate in the following:

- You will be asked to allow us to observe your visit with your clinician. This may be done by having a study team member present in the room during the appointment, by video-recording the visit with a small camera, or both. You, your clinician, and any visitors can turn off the recorder at any time. If there is a physical examination, the study team observing will step out of the room, and the recording device will be shut off.
- You may be asked for permission to take notes or record observations of things you say or do during your discussion with your clinician.
- Your clinician may use educational materials we have developed with you in your visit.
- We will determine whether your clinician will use the education materials by chance, like the flip of a coin, using a process called randomization. We will collect data from all participants in the same way (observations and/or recordings, surveys, and pharmacy records).
- You will be asked to complete a brief survey following your visit. We expect that this survey will take you approximately 5-10 minutes to complete.
- Another part of this study is looking at your pharmacy records. To get information for this part of the study, you will be asked to sign an authorization to release your pharmacy and medical records and billing records to Mayo Clinic.
- We may contact you to do a phone survey within one year after enrollment.

I permit the research team to keep the collected study data (including audio and video recordings) in a registry to conduct further analyses, future un-identified and IRB approved research, trainings, quality improvement and educational purposes, which includes sending data (and recordings) to external collaborators.

☐ Yes ☐ No Please initial here: ________ Date: ________

6. What are the possible risks or discomforts from being in this research study?

The risks to participating in this research study are minimal, which means that we do not believe they will be any different than what you would experience at a routine clinical encounter or during your daily life. Sometimes having a conversation observed or recorded can be distressing. If you wish to no longer be observed or recorded, you may ask the study team member observing to exit the room or turn off the recording device. Additionally, you may choose not to answer any questions you are uncomfortable answering on the post-visit survey.

7. What are the possible benefits from being in this research study?

Although you may not directly benefit from participating in this research study, there is a potential benefit to people in the future as a result of the information gathered in this research study.

8. What alternative do you have if you choose not to participate in this research study?

This study is only being done to gather information. You may choose not to take part in this study.

9. Will you be paid for taking part in this research study?

There is no compensation for participation in this study.

10. How will your privacy and the confidentiality of your records be protected?

Mayo Clinic is committed to protecting the confidentiality of information obtained about you in connection with this research study.

Your privacy is very important to us. We follow several procedures in order to protect your confidentiality. You will be given a unique study code that will be used instead of your name for the purposes of identifying and tracking you and all other participants in the study. If some of the information is reported in published medical journals or scientific discussions, it will be done in a way that does not directly identify you. The video files used in this study will be immediately transferred to secure and password-protected servers from which only authorized research personnel can conduct evaluations, and video files will be deleted from the recorder immediately following the visit. Similarly, all paper files will be stored in locked file cabinets in which only select personnel have access. We may send direct subject identifiers (voice, video, or audio recordings, images, and/or voice print data) to The Dartmouth Institute for Health Policy and Clinical Practice for data analysis and/or to external transcriptionists for transcription of the audio or video recordings.

During this research, information about your health will be collected. Under Federal law called the Privacy Rule, health information is private. However, there are exceptions to this rule, and you should know who may be able to see, use and share your health information for research and why they may need to do so. Information about you and your health cannot be used in this research study without your written permission. If you sign this form, it will provide that permission.

Health information may be collected about you from:

- Past, present and future medical records.
- Research procedures, including research office visits, tests, interviews and questionnaires.

Why will this information be used and/or given to others?

- To do the research.
- To report the results.
- To see if the research was done correctly.

If the results of this study are made public, information that identifies you will not be used.

Who may use or share your health information?

- Mayo Clinic research staff involved in this study.

With whom may your health information be shared?

- The Mayo Clinic Institutional Review Board that oversees the research.
- Other Mayo Clinic physicians involved in your clinical care.
- Researchers involved in this study at other institutions.
- Federal and State agencies (such as the Food and Drug Administration, the Department of Health and Human Services, the National Institutes of Health and other United States agencies) or government agencies in other countries that oversee or review research.

Is your health information protected after it has been shared with others?

Mayo Clinic asks anyone who receives your health information from us to protect your privacy; however, once your information is shared outside Mayo Clinic, we cannot promise that it will remain private and it may no longer be protected by the Privacy Rule.

Your Privacy Rights

You do not have to sign this form, but if you do not, you cannot take part in this research study.

If you cancel your permission to use or share your health information, your participation in this study will end and no more information about you will be collected; however, information already collected about you in the study may continue to be used.

If you choose not to take part or if you withdraw from this study, it will not harm your relationship with your own doctors or with Mayo Clinic.

You can cancel your permission to use or share your health information at any time by sending a letter to the address below:
Mayo Clinic
Office for Human Research Protection
ATTN: Notice of Revocation of Authorization
200 1st Street SW
Rochester, MN 55905

Alternatively, you may cancel your permission by emailing the Mayo Clinic Research Subject Advocate at: researchsubjectadvocate@mayo.edu

MAYO CLINIC

Name and Clinic Number

Approval Date: **January 18, 2017**
Not to be used after: **August 30, 2017**

Please be sure to include in your letter or email:

- The name of the Principal Investigator,
- The study IRB number and /or study name, and
- Your contact information.

Your permission lasts forever, unless you cancel it.

ENROLLMENT AND PERMISSION SIGNATURES

Your signature documents your permission to take part in this research.

	/ /	: AM/PM
Printed Name	Date	Time

____
Signature

Person Obtaining Consent

- I have explained the research study to the participant.
- I have answered all questions about this research study to the best of my ability.

	/ /	: AM/PM
Printed Name	Date	Time

____
Signature
