# Supplementary material for: Shared decision making for stroke prevention in atrial fibrillation: study protocol for a randomized controlled trial
Source: Trials. 2017 Sep 29;18:443. doi: 10.1186/s13063-017-2178-y (PMC5622521; doi:10.1186/s13063-017-2178-y)
Supplement: Supplementary file 3 — Clinician study information and informed consent form. (PDF 160 kb) [file 13063_2017_2178_MOESM3_ESM.pdf]

MAYO CLINIC

Name and Clinic Number

Approval Date: **January 18, 2017**
Not to be used after: **August 30, 2017**

RESEARCH PARTICIPANT CONSENT AND PRIVACY AUTHORIZATION FORM

Study Title: Shared Decision Making for Stroke Prevention in Atrial Fibrillation (SDM4Afib) (Clinician)

IRB#: 16-005409

Principal Investigator: Peter A. Noseworthy, M.D. and Colleagues

Please read this information carefully. It tells you important things about this research study. A member of our research team will talk to you about taking part in this research study. If you have questions at any time, please ask us.

Take your time to decide. Feel free to discuss the study with your family, friends, and healthcare provider before you make your decision.

To help you decide if you want to take part in this study, you should know:

- Taking part in this study is completely voluntary.
- You can choose not to participate.
- You are free to change your mind at any time if you choose to participate.
- Your decision won't cause any penalties or loss of benefits to which you're otherwise entitled.
- Your decision won't change the access to medical care you get at Mayo Clinic now or in the future if you choose not to participate or discontinue your participation.

For purposes of this form, Mayo Clinic refers to Mayo Clinic in Arizona, Florida and Rochester, Minnesota; Mayo Clinic Health System; and all owned and affiliated clinics, hospitals, and entities.

If you decide to take part in this research study, you will sign this consent form to show that you want to take part. We will give you a copy of this form to keep.

MAYO CLINIC

Name and Clinic Number

Approval Date: **January 18, 2017**
Not to be used after: **August 30, 2017**

CONTACT INFORMATION

You can contact …	At …	If you have questions about …
**Principal Investigator:**<br>Dr. Peter A. Noseworthy<br><br>**Study Team Contact:**<br>Cara Fernandez	**Phone:**<br>(507) 293-0175<br><br>**Phone:**<br>(507) 266-1893<br><br>**Institution Name and Address:**<br>Mayo Clinic<br>200 First Street SW<br>Rochester, MN 55905	▪ Study tests and procedures<br>▪ Research-related injuries or emergencies<br>▪ Any research-related concerns or complaints<br>▪ Withdrawing from the research study<br>▪ Materials you receive<br>▪ Research-related appointments
**Mayo Clinic Institutional Review Board (IRB)**	**Phone:**<br>(507) 266-4000<br><br>**Toll-Free:**<br>(866) 273-4681	▪ Rights of a research participant
**Research Subject Advocate (The RSA is independent of the Study Team)**	**Phone:**<br>(507) 266-9372<br><br>**Toll-Free:**<br>(866) 273-4681<br><br>**E-mail:**<br>researchsubjectadvocate@mayo.edu	▪ Rights of a research participant<br>▪ Any research-related concerns or complaints<br>▪ Use of your Protected Health Information<br>▪ Stopping your authorization to use your Protected Health Information
**Research Billing**	**Rochester, MN:** (507) 266-5670	▪ Billing or insurance related to this research study

A description of this clinical trial will be available on http://www.ClinicalTrials.gov, as required by U.S. Law. This Website will not include information that can identify you. At most, the Website will include a summary of the results. You can search this Website at any time.

1. Why are you being asked to take part in this research study?

We are inviting you to be part of this research study because you meet with patients who have atrial fibrillation.

2. Why is this research study being done?

This research study is being done to test the anticoagulation choice decision aid versus standard of care among patients with atrial fibrillation.

3. How long will you be in this research study?

You will be in this study until your last patient is enrolled. This study is expected to enroll participants for 2-3 years. You will be in this study until your last patient who agrees to participate is enrolled during this time frame.

4. What will happen to you while you are in this research study?

If you agree to participate, you will be asked to participate in the following:

- During trainings for the use of anticoagulation choice decision aid, you will be asked to allow us to observe the sessions. This may be done by having a study team member present in the room, by video-recording with small cameras, or both. Any member attending the training can turn off the recorders at any time. After trainings, you will be asked to fill out a brief survey as described below.
- You will be asked to complete a brief baseline survey before the first patient is enrolled. We expect that this survey will take less than 5 minutes to complete.
- Once a patient has been consented you will be informed which arm he or she is assigned to.

- Your patients will be randomized to one of two arms:
  - Intervention arm: use the atrial fibrillation decision aid to serve as a guide for conversation regarding treatment options for patients with atrial fibrillation
  - Control arm: discuss options for atrial fibrillation in the usual way
- If your patients are randomized to the decision aid arm, you will not be obligated to follow a script nor will you be required to use the decision aid with enrolled patients when you deem the tool inappropriate.
- You will be asked to allow us to observe your visit with your patient. This may be done by having a study team member present in the room during the appointment, by video-recording the visit with a small camera, or both. You, your patient, or any visitors can turn off the recorder at any time. If there is a physical examination, the study team observing the visit will step out of the room, and the recording device will be shut off.
- You may be asked for permission to take notes or record observations of things you say or do during your discussion with your patient.
- You will be asked to complete a brief survey following your visit with each patient enrolled. We expect that this survey will take you approximately 5 minutes to complete.

I permit the research team to keep the collected study data (including audio and video recordings) in a registry to conduct further analyses, future un-identified and IRB approved research, trainings, quality improvement and educational purposes, which includes sending data (and recordings) to external collaborators. (If you select no, your permission lasts until the end of this study.)

☐ Yes ☐ No Please initial here: ________ Date: ________

---

5. What are the possible risks or discomforts from being in this research study?

The risks to participating in this research study are minimal, which means that we do not believe they will be any different than what you would experience at a routine clinical encounter or during your daily life. Sometimes having a conversation observed or recorded can be distressing. If you wish to no longer be observed or recorded, you may ask the study team member observing to exit the room or turn off the recording device. Additionally, you may choose not to answer any questions you are uncomfortable answering on the post-visit survey.

Your participation in this study will not affect your current or future employment or be shared with your supervisor.

6. What are the possible benefits from being in this research study?

Although you may not directly benefit from participating in this research study, there is a potential benefit to people in the future as a result of the information gathered in this research study.

7. What alternative do you have if you choose not to participate in this research study?

This study is only being done to gather information. You may choose not to take part in this study.

8. Will you be paid for taking part in this research study?

There is no compensation for participation in this research study.

9. How will your privacy and the confidentiality of your records be protected?

Mayo Clinic is committed to protecting the confidentiality of information obtained about you in connection with this research study.

Your privacy is very important to us. We follow several procedures in order to protect your confidentiality. You will be given a unique study code that will be used instead of your name for the purposes of identifying and tracking you and all other participants in the study. If some of the information is reported in published medical journals or scientific discussions, it will be done in a way that does not directly identify you. The video files used in this study will be immediately transferred to secure and password-protected servers from which only authorized research

Name and Clinic Number

Approval Date: January 18, 2017
Not to be used after: August 30, 2017

personnel can conduct evaluations, and video files will be deleted from the recorder immediately following the visit. Similarly, all paper files will be stored in locked file cabinets in which only select personnel have access. We may send direct subject identifiers (voice, video, or audio recordings, images, and/or voice print data) to The Dartmouth Institute for Health Policy and Clinical Practice for data analysis and/or to external transcriptionists for transcription of the audio or video recordings.

Representatives from the Mayo Clinic Institutional Review Board (the committee that reviews, approves, and monitors research on human subjects) may inspect study records during internal auditing procedures. However, these individuals are required to keep all information confidential.

Your permission lasts forever, unless you cancel it.

ENROLLMENT AND PERMISSION SIGNATURES

Your signature documents your permission to take part in this research.

	/ /	: AM/PM
Printed Name	Date	Time

____
Signature

Person Obtaining Consent

- I have explained the research study to the participant.
- I have answered all questions about this research study to the best of my ability.

	/ /	: AM/PM
Printed Name	Date	Time

____
Signature
