# Supplementary material for: Shared decision making for stroke prevention in atrial fibrillation: study protocol for a randomized controlled trial
Source: Trials. 2017 Sep 29;18:443. doi: 10.1186/s13063-017-2178-y (PMC5622521; doi:10.1186/s13063-017-2178-y)
Supplement: Supplementary file 4 — The Anticoagulation Choice conversation tool. This conversation tool is an online tool intended for use by a patient and clinician together in a clinical encounter in which they are discussing how to respond to the patient’s risk of stroke due to nonvalvular atrial fibrillation (AF). The tool is intended to provide a supportive structure and information that patient and clinician may draw on during clinical conversations in various settings e.g. primary care, emergency department, cardiology and other specialty practices. Details on the full developmental process of the ANTICOAGULATION CHOICE conversation tool is described elsewhere (Hargraves et al, The Lion and the Snake: Design in Support of Shared Decision Making in Atrial Fibrillation. Manuscript in preparation). (PDF 742 kb) [file 13063_2017_2178_MOESM4_ESM.pdf]

Anticoagulation Choice
Decision Aid

This conversation tool is an online tool intended for use by a patient and clinician together in a clinical encounter in which they are discussing how to respond to the patient's risk of stroke due to nonvalvular atrial fibrillation (AF). The tool is intended to provide a supportive structure and information that patient and clinician may draw on during clinical conversations in various settings e.g. primary care, emergency department, cardiology and other specialty practices.

The tool begins by orienting patient and clinician to the conversation in which it will be used—the management of AF. The tool then moves to a CHA_2DS_2-VASc calculator that is used to review the medical situation with the patient. Based on the calculation, the tool then presents the patient's current risk of stroke without anticoagulation over 5 years (default) or 1 year (optional) through a prose description of natural frequencies and a pictogram. This presentation works to develop an initial appreciation of the problem facing patient and clinician—the patient's risk of stroke.

Next, the patient's risk of stroke with anticoagulation (without identifying a particular agent) is presented again through prose description and pictograph. This presentation works to introduce anticoagulation as an option, or hypothesis, for meeting the threat of AF.

Patient and clinician can then make an initial appraisal of this hypothesis and if this appraisal suggests that anticoagulation is worth considering then the tool moves on to introduce two approaches to anticoagulation—Warfarin and Direct Anticoagulants.

Whether and how to anticoagulate as an option or hypothesis is further tested in conversation with support from the tool through consideration of the practical issues involved in bringing anticoagulation into the patient's life (issues of bleeding with anticoagulation, anticoagulation and INR testing in the patient's routine, reversal of anticoagulation, cost, diet and other medications).

These issues are presented through panels that compactly orient patient and clinician to the implications of the two approaches to anticoagulation. The issue of bleeding is supported through four levels of discussion—not all of which need be explored. The first level describes the bleeding events associated with anticoagulation ranging from bruising to the need for emergency treatment for life-threatening bleeding. As the latter is a very serious occurrence, the second level qualifies what this threat means. It does this through presentation of the increased occurrence with anticoagulation, for the average person (HAS-BLED 2), of emergency treatment for bleeding through a prose description and a pictogram. The third level invites reflection on risk factors in the patient's environment and activities. If warranted, a fourth level supports consideration of the patient medical factors that may affect the patient's bleeding risk. This discussion may be handled through review of the HAS-BLED factors, or through use of the included HAS-BLED calculator to determine if the patient is at lower than average risk (HAS-BLED<2], average risk, or higher than average risk.

The tool closes in the decision of whether to respond to this patient's threat of stroke through anticoagulation or not, and if anticoagulation is selected, how anticoagulation will be brought into the patient's life (Warfarin, or a direct anticoagulant). This final screen also contains an editable description of the decision for the medical record, along with the option to print a summary report of the discussion for the patient.

Details on the full developmental process of the ANTICOAGULATION CHOICE conversation tool is described elsewhere.

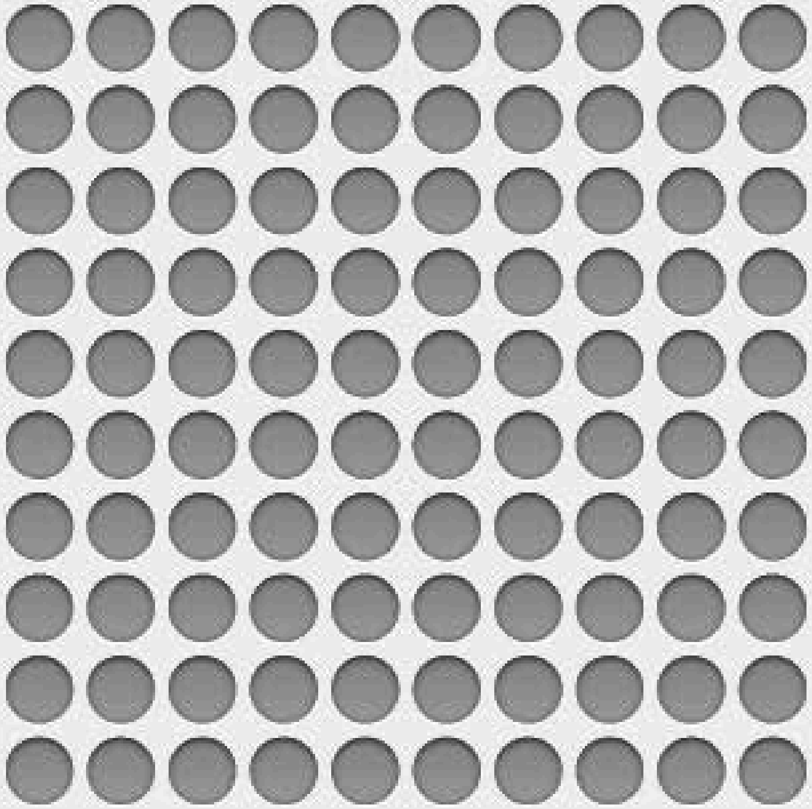

Welcome to the **Anticoagulation Choice** Decision Aid.

This tool will help you and your doctor discuss how to manage your Atrial Fibrillation

Let's get started

Caution: This application is for use exclusively during the clinical encounter with your clinician

LEGAL CONDITIONS AND TERMS OF USE APPLICABLE TO ALL USERS OF THIS SITE. ANY USE OF THIS SITE CONSTITUTES YOUR AGREEMENT TO THESE TERMS AND CONDITIONS OF USE.

© 1998-2015 Mayo Foundation for Medical Education and Research. All rights reserved.

MAYO CLINIC **Anticoagulation Choice**
Decision Aid

- 1 Year Risk
- 5 Year Risk (selected)

Medical Situation | Risk of Stroke | Anticoagulation | Issues

CHA_2DS_2-VASC **2**

To begin, let's review your medical situation

Gender	M / F (F selected)
Age	68
History of Hypertension	No
Congestive Heart Failure	No
Stroke / TIA / Thromboembolism	No
History of Vascular Disease	No
Diabetes Mellitus	No

Continue to consider your risk of stroke

Credits & Contacts

Privacy policy (Updated October, 2014) Terms and conditions of use policy (Updated February 6, 2015)

LEGAL CONDITIONS AND TERMS OF USE APPLICABLE TO ALL USERS OF THIS SITE. ANY USE OF THIS SITE CONSTITUTES YOUR AGREEMENT TO THESE TERMS AND CONDITIONS OF USE.

© 1998-2015 Mayo Foundation for Medical Education and Research. All rights reserved.

MAYO CLINIC

Anticoagulation Choice
Decision Aid

- 1 Year Risk
- 5 Year Risk (selected)

Medical Situation | **Risk of Stroke** | Anticoagulation | Issues

CHA2DS2-VASC **2**

Over the next 5 years

87 people will have no stroke

6 people will have a fatal or disabling stroke

7 people will have a non-disabling stroke

Current Risk
of Stroke without Anticoagulation

In 100 people like you who **are not** taking an anticoagulant

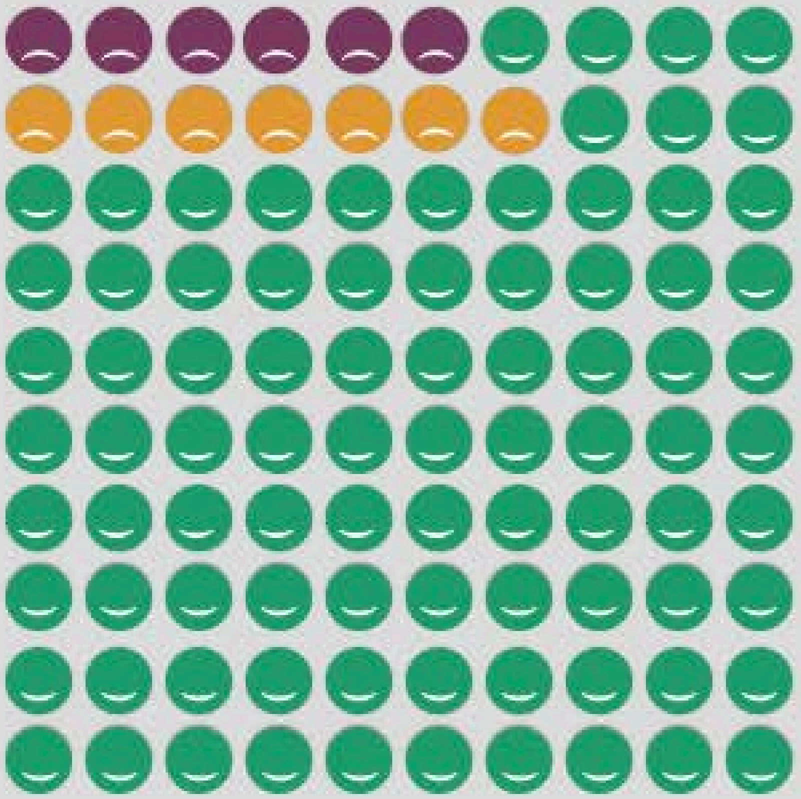

With Anticoagulation

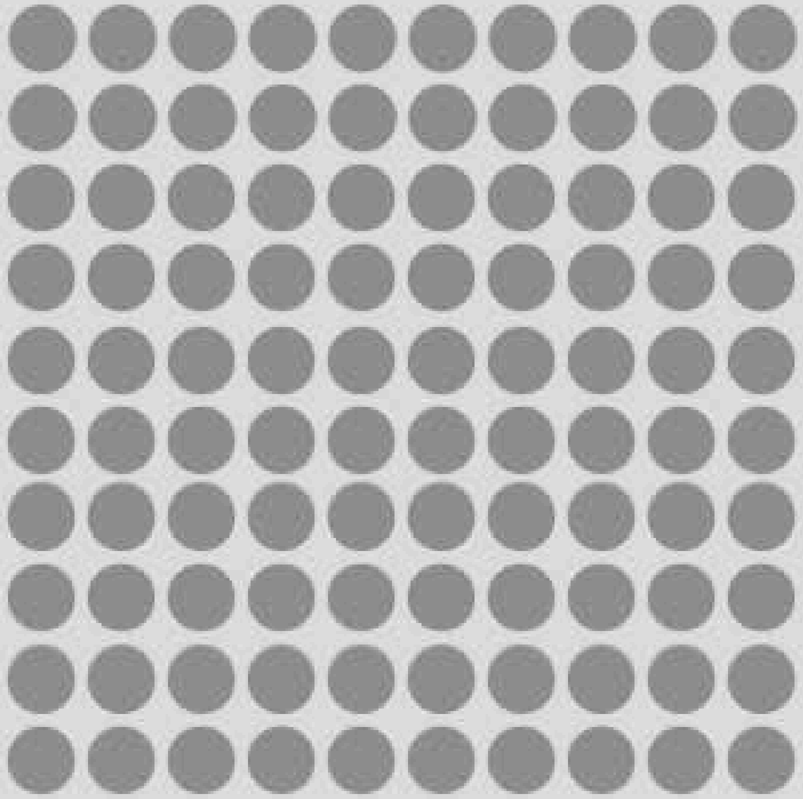

LEGAL CONDITIONS AND TERMS OF USE APPLICABLE TO ALL USERS OF THIS SITE. ANY USE OF THIS SITE CONSTITUTES YOUR AGREEMENT TO THESE TERMS AND CONDITIONS OF USE.

© 1998-2015 Mayo Foundation for Medical Education and Research. All rights reserved.

1 Year Risk
5 Year Risk

Medical Situation | Risk of Stroke | **Anticoagulation** | Issues

CHA_2DS_2-VASC **2**

Current Risk
of Stroke without Anticoagulation

Over the next 5 years

87 people will have no stroke

6 people will have a fatal or disabling stroke

7 people will have a non-disabling stroke

In 100 people like you who **are not** taking an anticoagulant

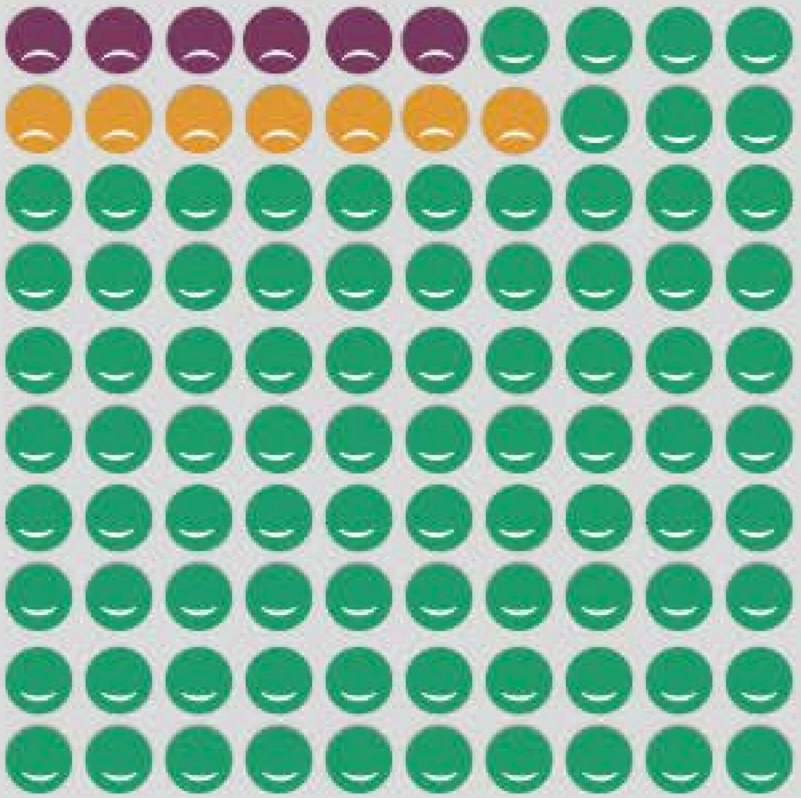

Future Risk
of Stroke with Anticoagulation

In 100 people like you who **are** taking an anticoagulant

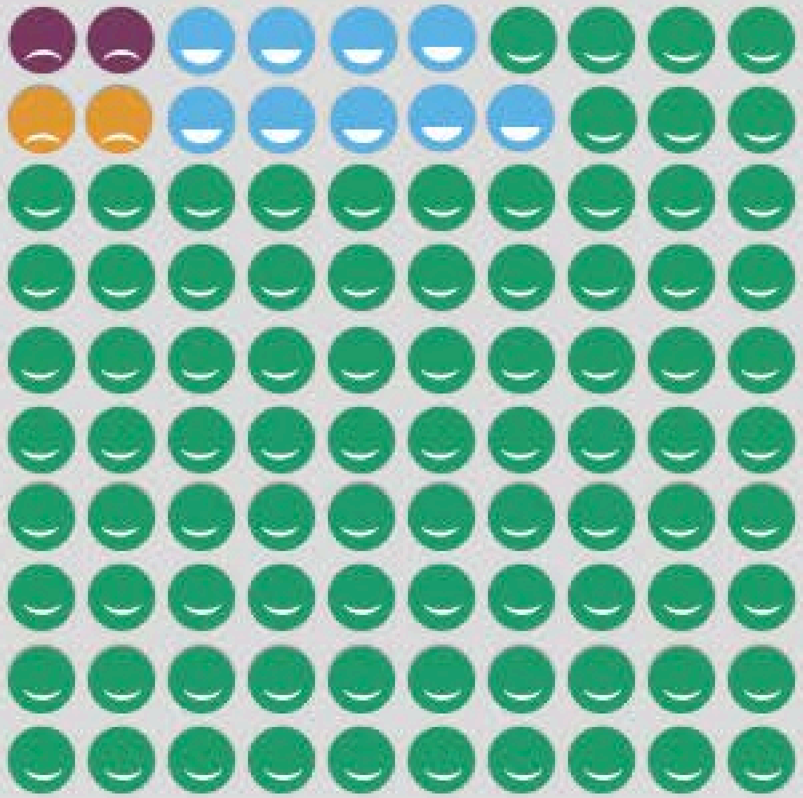

Over the next 5 years

96 people will have no stroke

2 people will have a fatal or disabling stroke

2 people will have a non-disabling stroke

9 people will avoid a stroke by taking anticoagulation

LEGAL CONDITIONS AND TERMS OF USE APPLICABLE TO ALL USERS OF THIS SITE. ANY USE OF THIS SITE CONSTITUTES YOUR AGREEMENT TO THESE TERMS AND CONDITIONS OF USE.

© 1998-2015 Mayo Foundation for Medical Education and Research. All rights reserved.

MAYO CLINIC

Anticoagulation Choice
Decision Aid

○ 1 Year Risk
● 5 Year Risk

Medical Situation | Risk of Stroke | Anticoagulation | **Issues**

CHA$_2$DS$_2$-VASC **2**

- Risk of Bleeding
- Anticoagulation Routine
- Reversing Anticoagulation
- Cost
- Diet & Medication Interactions

Diet & Medication Interactions

Warfarin ++++

Requires a stable diet.

Interacts with common foods, medications, and supplements.

Direct Anticoagulants +

Few interactions with food or medications.

Can you maintain the stable diet that Warfarin requires?

Anticoagulation Routine

Warfarin

Once daily | Regular blood tests

Direct Anticoagulants

Apixaban	Eliquis	AM	PM
Dabigatran	Pradaxa	AM	PM
Edoxaban	Savaysa	Once daily	
Rivaroxaban	Xarelto	Once daily	

Are you available to do the regular blood tests that Warfarin requires?

Credits & Contacts

Privacy policy (Updated October, 2014) Terms and conditions of use policy (Updated February 6, 2015)

LEGAL CONDITIONS AND TERMS OF USE APPLICABLE TO ALL USERS OF THIS SITE. ANY USE OF THIS SITE CONSTITUTES YOUR AGREEMENT TO THESE TERMS AND CONDITIONS OF USE.

© 1998-2015 Mayo Foundation for Medical Education and Research. All rights reserved.

Risk of Bleeding

When taking an Anticoagulant you may...

- bruise more easily
- bleed more easily
- require emergency treatment for life-threatening bleeding

Risk of needing emergency treatment

Reversing Anticoagulation

Warfarin

Medications to reverse the effects of Warfarin are

commonly available

Direct Anticoagulants

Medications to reverse the effects of Direct Anticoagulants are

not commonly available

LEGAL CONDITIONS AND TERMS OF USE APPLICABLE TO ALL USERS OF THIS SITE. ANY USE OF THIS SITE CONSTITUTES YOUR AGREEMENT TO THESE TERMS AND CONDITIONS OF USE.

© 1998-2015 Mayo Foundation for Medical Education and Research. All rights reserved.

MAYO CLINIC

Anticoagulation Choice
Decision Aid

1 Year Risk
5 Year Risk

Medical Situation | Risk of Stroke | Anticoagulation | **Issues**

CHA_2DS_2-VASC **2**
HAS-BLED **1**

Risk of Bleeding

Anticoagulation Routine

Reversing Anticoagulation

Cost

Diet & Medication Interactions

Five Year Risk of Bleeding

For Average Risk

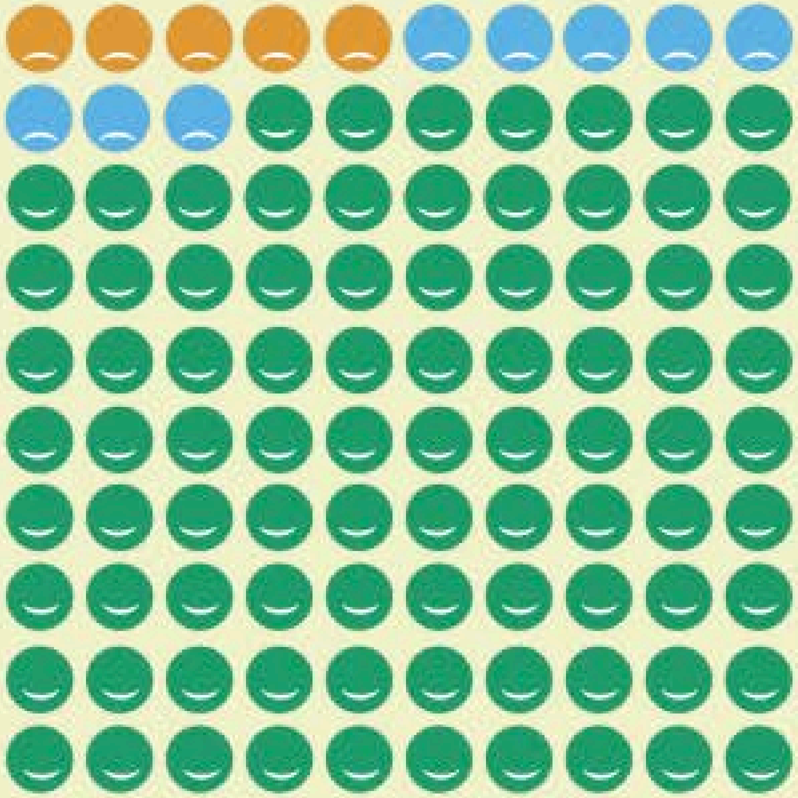

Exit

In 100 people, over the next 5 years…

87 people will not need emergency treatment for bleeding

5 will need emergency treatment whether they take an anticoagulant or not

8 more people will need emergency treatment because they take an anticoagulant

Some bleeds are fatal

Your risk may be different
Discuss your risk factors

Credits & Contacts

Privacy policy (Updated October, 2014) Terms and conditions of use policy (Updated February 6, 2015)

LEGAL CONDITIONS AND TERMS OF USE APPLICABLE TO ALL USERS OF THIS SITE. ANY USE OF THIS SITE CONSTITUTES YOUR AGREEMENT TO THESE TERMS AND CONDITIONS OF USE.

© 1998-2015 Mayo Foundation for Medical Education and Research. All rights reserved.

1 Year Risk
5 Year Risk

Medical Situation | Risk of Stroke | Anticoagulation | **Issues**

CHA$_2$DS$_2$-VASC **2**
HAS-BLED **1**

Risk of Bleeding

Anticoagulation Routine

Reversing Anticoagulation

Cost

Diet & Medication Interactions

Five Year Risk of Bleeding

Risk factors in your day-to-day life

Are there activities at work, home or during recreation where you might fall or hurt yourself?

Exit

Discuss your medical risk factors

LEGAL CONDITIONS AND TERMS OF USE APPLICABLE TO ALL USERS OF THIS SITE. ANY USE OF THIS SITE CONSTITUTES YOUR AGREEMENT TO THESE TERMS AND CONDITIONS OF USE.

© 1998-2015 Mayo Foundation for Medical Education and Research. All rights reserved.

MAYO CLINIC

Anticoagulation Choice
Decision Aid

( ) 1 Year Risk
(•) 5 Year Risk

Medical Situation | Risk of Stroke | Anticoagulation | **Issues**

CHA_2DS_2-VASC **2**
HAS-BLED **1**

- Risk of Bleeding
- Anticoagulation Routine
- Reversing Anticoagulation
- Cost
- Diet & Medication Interactions

Risk of Bleeding

Risk factors in your medical situation

Age	
Uncontrolled Hypertension	No
Renal Disease	No
Liver Disease	No
History of Stroke	No
Prior, or Predisposition to Bleeding	No
Unstable or High INR	No
Medication Predisposing Bleeding	No
More than 8 Drinks per Week	No

Exit

Medical factors associated with bleeding may put you at

- Lower than Average Risk
- Average Risk
- Higher than Average Risk

Privacy policy (Updated October, 2014) Terms and conditions of use policy (Updated February 6, 2015)
LEGAL CONDITIONS AND TERMS OF USE APPLICABLE TO ALL USERS OF THIS SITE. ANY USE OF THIS SITE CONSTITUTES YOUR AGREEMENT TO THESE TERMS AND CONDITIONS OF USE.
© 1998-2015 Mayo Foundation for Medical Education and Research. All rights reserved.

MAYO CLINIC

Anticoagulation Choice
Decision Aid

○ 1 Year Risk
● 5 Year Risk

Medical Situation | Risk of Stroke | Anticoagulation | **Issues**

CHA₂DS₂-VASC **2**
HAS-BLED **3**

Risk of Bleeding

Anticoagulation Routine

Reversing Anticoagulation

Cost

Diet & Medication Interactions

Five Year Risk of Bleeding

Risk factors in your medical situation

Risk factor	Value
Age	68
Uncontrolled Hypertension	Yes
Renal Disease	No
Liver Disease	Yes
History of Stroke	No
Prior, or Predisposition to Bleeding	No
Unstable or High INR	No
Medication Predisposing Bleeding	No
More than 8 Drinks per Week	No

Exit

Higher than Average Risk

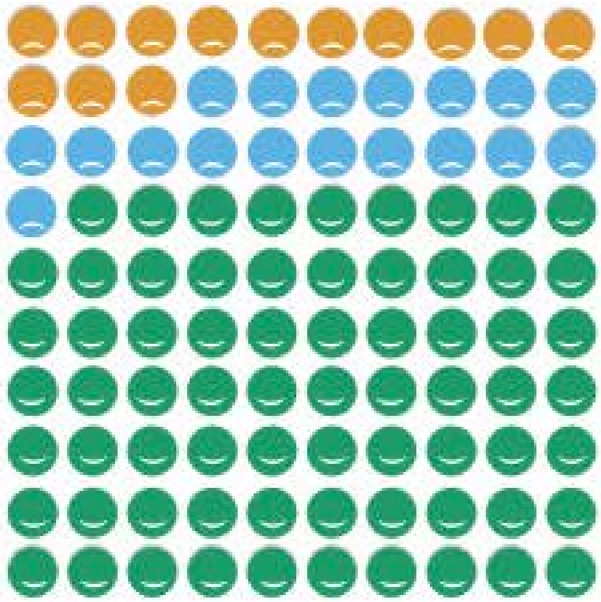

Over 5 years
18 more people out of 100 will need emergency treatment for bleeding because they take an anticoagulant
Some bleeds are fatal

serious bleed **without** anticoagulation
additional serious bleed **with** anticoagulation

Credits & Contacts

Privacy policy (Updated October, 2014) Terms and conditions of use policy (Updated February 6, 2015)

LEGAL CONDITIONS AND TERMS OF USE APPLICABLE TO ALL USERS OF THIS SITE. ANY USE OF THIS SITE CONSTITUTES YOUR AGREEMENT TO THESE TERMS AND CONDITIONS OF USE.

© 1998-2015 Mayo Foundation for Medical Education and Research. All rights reserved.
