# Supplementary material for: Shared decision making for stroke prevention in atrial fibrillation: study protocol for a randomized controlled trial
Source: Trials. 2017 Sep 29;18:443. doi: 10.1186/s13063-017-2178-y (PMC5622521; doi:10.1186/s13063-017-2178-y)
Supplement: Supplementary file 5 — Patient post-encounter survey. (PDF 1449 kb) [file 13063_2017_2178_MOESM5_ESM.pdf]

MAYO
CLINIC

Shared Decision Making for Stroke Prevention in Atrial Fibrillation

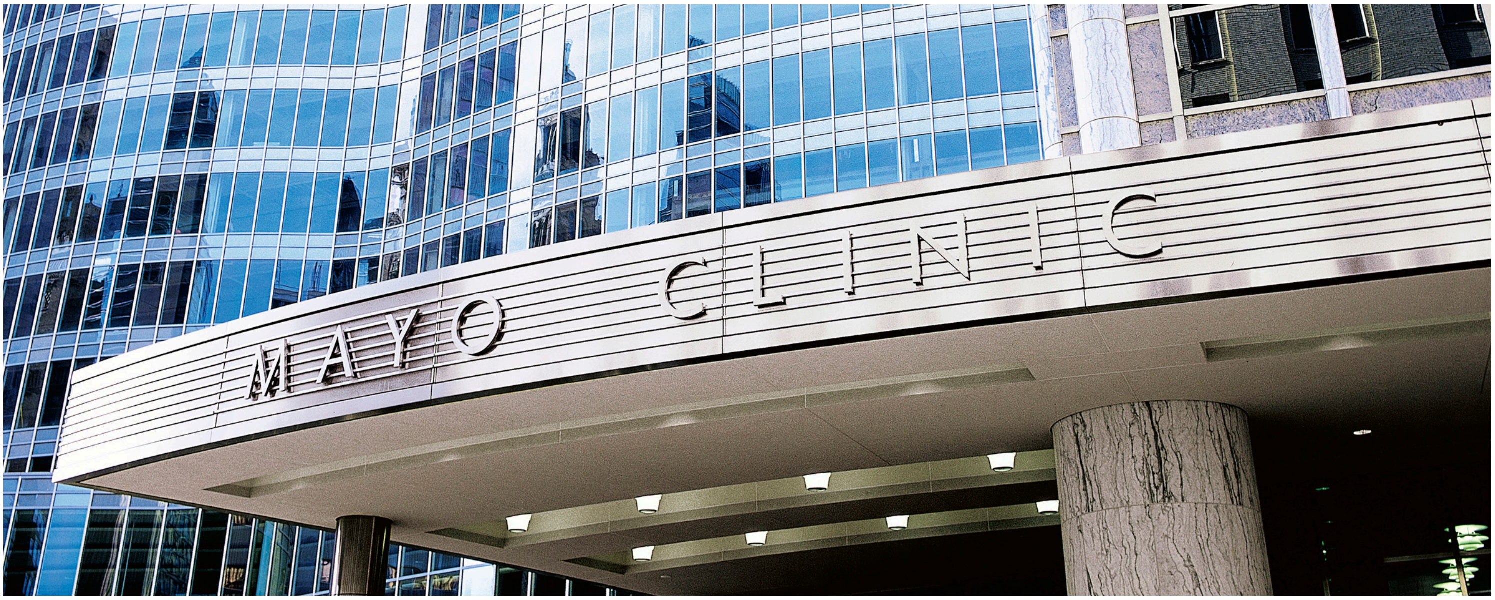

Survey Research Center

Thank you for taking the time to complete this survey. Your answers are important to us. Please take the time to read and answer each question carefully by marking the box or providing the response that best represents your answers. Your responses are confidential and your clinician will not see your answers.

After completing this survey, please return it to the clinical staff that gave it to you. Please feel free to ask any questions you have while completing the survey.

Thank you.

Patient ID: __ __ __ __ __ __ __

INSTRUCTIONS: PLEASE CHECK THE APPROPRIATE BOX OR FILL IN THE BLANK AS INDICATED.

1. **Today's Date:** __ __/__ __/__ __ __ __
   Month Day Year

Below are some statements about blood thinners (anticoagulation medicines). Please show whether you think they are correct (True), not correct (False), or you are unsure (Do not know). This is not a test - what is important is that your answers reflect what you think is true about blood thinners (anticoagulation medicines).

	True	False	Do not know
2. **Taking blood thinners can lower my risk of stroke.**	1 ☐	2 ☐	3 ☐
3. **Taking blood thinners can increase my risk of bleeding.**	1 ☐	2 ☐	3 ☐
4. **Some, but not all, blood thinners require me to get periodic blood tests.**	1 ☐	2 ☐	3 ☐
5. **Blood thinners can be used to treat a stroke if I have one.**	1 ☐	2 ☐	3 ☐
6. **Blood thinners must be taken daily.**	1 ☐	2 ☐	3 ☐
7. **I can safely eat whatever I want when taking Warfarin.**	1 ☐	2 ☐	3 ☐

8. **Of 100 people like me, the number expected to have a disabling or fatal stroke in the next year is: Provide a value between 0 - 100.**

   __ __ __ 1 ☐ I don't know

9. **Which decision did you and your clinician make today about taking a blood thinner?** (Please mark one.)

   1 ☐ Start or continue taking the blood thinner Warfarin
   2 ☐ Start or continue taking one of the blood thinners known as direct anticoagulants: Apixaban (Eliquis), Dabigatran (Pradaxa), Edoxaban (Savaysa), or Rivaroxaban (Xarelto)
   3 ☐ Stop taking or not start any blood thinner
   4 ☐ Make a decision at some other time
   5 ☐ Other decision, please specify: ______________________________

10. **Was the cost of the blood thinner (anticoagulant medicine) a factor in your decision?**

   1 ☐ No, cost did not matter
   2 ☐ Yes, cost was one factor I considered in my decision
   3 ☐ Yes, cost was the sole factor in my decision

Now, thinking about the decision you made with your clinician about blood thinners today, please look at the following comments that some people have made when deciding about blood thinners to lower their risk of stroke. Please show how strongly you agree or disagree with these comments by checking the box that indicates your level of agreement.

	Strongly agree	Agree	Neither agree nor disagree	Disagree	Strongly disagree
**11. I know which options are available to me.**	1 ☐	2 ☐	3 ☐	4 ☐	5 ☐
**12. I know the benefits of each option.**	1 ☐	2 ☐	3 ☐	4 ☐	5 ☐
**13. I know the risks and side effects of each option.**	1 ☐	2 ☐	3 ☐	4 ☐	5 ☐
**14. I am clear about which benefits matter most to me.**	1 ☐	2 ☐	3 ☐	4 ☐	5 ☐
**15. I am clear about which risks and side effects matter most to me.**	1 ☐	2 ☐	3 ☐	4 ☐	5 ☐
**16. I am clear about which is more important to me (the benefits or the risks and side effects).**	1 ☐	2 ☐	3 ☐	4 ☐	5 ☐
**17. I have enough support from others to make a choice.**	1 ☐	2 ☐	3 ☐	4 ☐	5 ☐
**18. I am choosing without pressure from others.**	1 ☐	2 ☐	3 ☐	4 ☐	5 ☐
**19. I have enough advice to make a choice.**	1 ☐	2 ☐	3 ☐	4 ☐	5 ☐
**20. I am clear about the best choice for me.**	1 ☐	2 ☐	3 ☐	4 ☐	5 ☐
**21. I feel sure about what to choose.**	1 ☐	2 ☐	3 ☐	4 ☐	5 ☐
**22. This decision is easy for me to make.**	1 ☐	2 ☐	3 ☐	4 ☐	5 ☐
**23. I feel I have made an informed choice.**	1 ☐	2 ☐	3 ☐	4 ☐	5 ☐
**24. I expect to stick with my decision.**	1 ☐	2 ☐	3 ☐	4 ☐	5 ☐
**25. The decision shows what is important to me.**	1 ☐	2 ☐	3 ☐	4 ☐	5 ☐
**26. I am satisfied with my decision.**	1 ☐	2 ☐	3 ☐	4 ☐	5 ☐

Thinking of the conversation you just had with your clinician about blood thinners (anticoagulation medicines), please select the most appropriate response to each item below:

	Yes, definitely	Yes, somewhat	No
**27. Did your clinician explain things in a way that was easy to understand?**	1 ☐	2 ☐	3 ☐
**28. Did your clinician listen carefully to you?**	1 ☐	2 ☐	3 ☐
**29. Did your clinician show respect for what you had to say?**	1 ☐	2 ☐	3 ☐

30. Would you recommend the way that you and your clinician shared information about your blood thinners (anticoagulation medicines) to other patients?

1 ☐	2 ☐	3 ☐	4 ☐	5 ☐	6 ☐	7 ☐
Yes, I would strongly recommend it			Not sure whether to recommend it or not			No, 1 would strongly recommend against it

31. In general, would you say your health is:

1 ☐ Excellent 2 ☐ Very good 3 ☐ Good 4 ☐ Fair 5 ☐ Poor

32. How many different medicines in total, prescription and over-the-counter, do you take per day? (Please count a medicine taken several times per day as one medicine.)

__ __ Number of different medicines per day 1 ☐ I do not take any medicines

33. Are you currently taking any of the following medications?

1 ☐ Aspirin
2 ☐ NSAIDS (ibuprofen/Advil, naproxen/Aleve, indomethacin/Indocin, diclofenac/Voltaren)
3 ☐ Antiplatelet agents (clopidogrel/Plavix, ticagrelor/Brilinta, prasugrel/Effient)
4 ☐ No, I do not take any of these medications currently.

34. Do you have a medical condition that increases your chance of bleeding?
(i.e., anemia, history of gastric or intestinal ulcers, diverticulosis, hemophelia)

1 ☐ No   2 ☐ Yes

If yes, which of the bleeding conditions do you have?
(Mark all that apply.)

1 ☐ Anemia
1 ☐ History of gastric or intestinal ulcers
1 ☐ Diverticulosis
1 ☐ Inherited condition of the blood system such as hemophilia
1 ☐ Other, please specify: ______________________________

35. In a typical week (7-day period), how many alcoholic drinks do you consume?
One drink is 12 ounces of beer, 5 ounces of wine or 1.5 ounces of hard alcohol.

__ __ Drinks per week

36. Have you ever had a bleeding event that required hospitalization?

1 ☐ No   2 ☐ Yes

If yes,

When was the most recent event: __ __/__ __ __ __
Month Year

What was the nature of the event: ______________________________

37. Have you ever suffered a fall that has resulted in a major bleed, broken bone, head injury, or required hospitalization?

1 ☐ No   2 ☐ Yes

If yes, what was the nature of the most recent fall?

____

38. Do you reside in a long-term care facility (i.e., nursing home)?

1 ☐ No   2 ☐ Yes

39. Do you receive your primary healthcare needs at another healthcare organization than this one?

1 ☐ No   2 ☐ Yes

FOR EACH OF THE FOLLOWING QUESTIONS, PLEASE CHECK THE BOX THAT <u>BEST REFLECTS YOUR ANSWER.</u>

40. How confident are you filling out medical forms by yourself?

1 ☐ Not at all 2 ☐ A little bit 3 ☐ Somewhat 4 ☐ Quite a bit 5 ☐ Extremely

41. When reading the newspaper, how <u>helpful</u> do you find tables and graphs that are parts of a story?

1 ☐	2 ☐	3 ☐	4 ☐	5 ☐	6 ☐
Not at all helpful					Extremely helpful

42. When people tell you the chance of something happening, do you prefer that they use <u>words</u> ("it rarely happens") or <u>numbers</u> ("there's a 1% chance")?

1 ☐	2 ☐	3 ☐	4 ☐	5 ☐	6 ☐
Always prefer words					Always prefer numbers

43. When you hear a weather forecast, do you prefer predictions using <u>percentages</u> (e.g., "there will be a 20% chance of rain today") or predictions using only <u>words</u> (e.g., "there is a small chance of rain today")?

1 ☐	2 ☐	3 ☐	4 ☐	5 ☐	6 ☐
Always prefer percentages					Always prefer words

44. How <u>often</u> do you find numerical information to be useful?

1 ☐	2 ☐	3 ☐	4 ☐	5 ☐	6 ☐
Never					Very often

Some questions about you . . .

45. Are you currently: (Please mark one.)

1 ☐ Married
2 ☐ Living with someone in a marriage-like relationship
3 ☐ Separated
4 ☐ Divorced
5 ☐ Widowed
6 ☐ Never married

46. What is the highest level of schooling you have completed? (Please mark one.)

1 ☐ Some high school or less
2 ☐ High school graduate or GED
3 ☐ Some college or associate's degree (including community college) or vocational technical, or business school degree
4 ☐ Four-year college graduate (bachelor's degree)
5 ☐ Graduate or professional school degree
6 ☐ Other, please specify: ______________________________

47. Which of the following categories best describes your household income last year before taxes?

1 ☐ Less than $20,000
2 ☐ $20,000 to $29,999
3 ☐ $30,000 to $39,999
4 ☐ $40,000 to $59,999
5 ☐ $60,000 to $79,999
6 ☐ $80,000 to $99,999
7 ☐ $100,000 to $149,999
8 ☐ $150,000 to $199,999
9 ☐ $200,000 or more

48. Are you of Hispanic, Latino, or Spanish origin?

1 ☐ Yes (Mexican, Mexican American, Chicano, Puerto Rican, Cuban, Other)
2 ☐ No, not Hispanic, Latino, or Spanish origin

49. What is your race? (Mark all that apply.)

1 ☐ White
1 ☐ Black or African American
1 ☐ American Indian or Alaska Native
1 ☐ Asian
1 ☐ Native Hawaiian or other Pacific Islander
1 ☐ Other, please specify: ______________________________

Thank you for completing the survey!

Please return it to the clinical support staff that asked you to complete it.

Questions 11-26: AM O'Connor, Decisional Conflict Scale. Copyright © 1993 [updated 2005]. Available from www.ohri.ca/decisionaid

MAYO CLINIC | 200 First Street SW | Rochester, MN 55905 | mayoclinic.org

©2016 Mayo Foundation for Medical Education and Research. All rights reserved. MAYO, MAYO CLINIC and the triple-shield Mayo logo are trademarks and service marks of MFMER.

MC4269-000

(THIS PAGE IS FOR SURVEY RESEARCH TRACKING AND FILING PURPOSES ONLY)

SHARED DECISION MAKING FOR STROKE PREVENTION IN ATRIAL FIBRILLATION (Third)

Investigator: Megan Branda

Version as of:
September 30, 2016 ddg
October 26, 2016 wld
November 11, 2016 ddg
November 30, 2016 wld

Coding Check:

Include:
