# Supplementary material for: Shared decision making for stroke prevention in atrial fibrillation: study protocol for a randomized controlled trial
Source: Trials. 2017 Sep 29;18:443. doi: 10.1186/s13063-017-2178-y (PMC5622521; doi:10.1186/s13063-017-2178-y)
Supplement: Supplementary file 6 — Clinician post-encounter survey. (PDF 42 kb) [file 13063_2017_2178_MOESM6_ESM.pdf]

Clinician Study ID: ___________________

Patient Study ID: ___________________

Today's Date: __ __/__ __/__ __ __ __
Month Day Year

Shared decision making for stroke prevention in atrial fibrillation (SDM4Afib)

Thank you for taking the time to complete this survey. Your answers are very important to us. Thinking about the conversation you just had with your patient about anticoagulation medications, please read and answer each question. Your responses are confidential.

1. **What decision did you and your patient make today regarding anticoagulation medication?**

   1 ☐ To start or continue on Warfarin
   2 ☐ To start or continue on Apixaban (Eliquis) or
   Dabigatran (Pradaxa) or
   Edoxaban (Savaysa) or
   Rivaroxaban (Xarelto)
   3 ☐ To not take an anticoagulation medication
   4 ☐ To start taking Aspirin or other antiplatelet agent for the purpose of stroke prevention
   5 ☐ To make the decision at some other time
   6 ☐ No discussion about anticoagulation medication took place
   7 ☐ Other, please specify: ____

2. **To what extent did you feel satisfied with the discussions you just had with your patient about taking an anticoagulant?**

1 ☐	2 ☐	3 ☐	4 ☐	5 ☐	6 ☐
NA no discussion	Not at all satisfied		Somewhat satisfied		Completely satisfied

3. **Would you recommend to other clinicians the way you and your patient worked together today to make a decision about taking an anticoagulant?**

1 ☐	2 ☐	3 ☐	4 ☐	5 ☐	6 ☐	7 ☐	8 ☐
NA No discussion	No, I would strongly recommend against it			Not sure whether to recommend it or not			Yes, I would strongly recommend it

(THIS PAGE IS FOR SURVEY RESEARCH TRACKING AND FILING PURPOSES ONLY)

SHARED DECISION MAKING FOR STROKE PREVENTION IN ATRIAL FIBRILLATION (Second)

Investigator: Megan Branda

Version as of:
September 30, 2016 ddg
October 24, 2016 wld
November 15, 2016 ddg
November 23, 2016 WLD
November 30, 2016 WLD

Coding Check:

Include:
